# Impact of WeChat public platform-based education and training on community nurses’ attitudes toward death and death coping skills: a prospective multicenter single-group pre-post study

**DOI:** 10.3389/fpubh.2026.1839677

**Published:** 2026-07-22

**Authors:** Chi Zhang, Qiyan Peng, Xinting Yang, Xiaoling Li

**Affiliations:** 1 Department of Cardiology, West China Hospital, Sichuan University/ West China School of Nursing, Sichuan University, Chengdu, China; 2 School of Nursing and Health Care, Leshan Vocational & Technical College, Leshan, China; 3 Wound Therapy Center, West China Hospital, Sichuan University/ West China School of Nursing, Sichuan University, Chengdu, China; 4 West China Hospital, West China School of Nursing, Sichuan University, Chengdu, China

**Keywords:** attitude to death, before-and-after study, community health nursing, coping skills, health education, mobile applications (WeChat)

## Abstract

**Objective:**

Death education is relevant to end-of-life nursing care, yet community nurses in primary care often receive little structured preparation in this area. This study developed a WeChat public platform-based death education program through expert consultation and evaluated changes in community nurses’ attitudes toward death and death coping skills before and after the program.

**Methods:**

We conducted a prospective before-and-after study in 10 community health centers in Chengdu, China, using a single-group design. The program was developed through two rounds of Delphi consultation and delivered through the WeChat public platform over 2 months. Participants completed the Death Attitude Profile-Revised (DAP-R) and Coping with Death Scale (CDS) before and after training. For paired pre-post comparisons, variables with normally distributed difference scores were examined with paired-samples *t*-tests, and variables with non-normal difference scores were examined with Wilcoxon signed-rank tests.

**Results:**

Seven experts completed both Delphi rounds, with response rates of 100% and authority coefficients of 0.922 and 0.929, respectively. The final program comprised five modules and 32 items. A total of 129 community nurses completed the two-month WeChat-based death education program and both the pretest and posttest assessments. After training, fear of death and death avoidance scores were lower (both *p* < 0.001). In contrast, natural acceptance (*p* = 0.054), approach acceptance (*p* = 0.339), and escape acceptance (*p* = 0.082) showed no statistically significant change. The CDS mean item score increased from 4.02 ± 0.67 to 4.20 ± 0.60 (*p* < 0.001; *r* = 0.72). Self-acceptance of death, death-coping ability, expression of death-related thoughts, life reflection, and ability to discuss death improved (*p* < 0.001). Funeral handling ability (*p* = 0.119) and coping with loss (*p* = 0.141) did not change significantly.

**Conclusion:**

The WeChat-based program was associated with reduced fear and avoidance of death and improvements in several dimensions of perceived death-coping ability. The absolute increase in the overall CDS mean item score was modest, and the single-group design prevents causal attribution. Controlled studies with longer follow-up and performance-based outcomes are needed.

## 1 Introduction

The latest China Statistical Yearbook shows that China’s population aged 65 years or above reached 200 million by the end of 2022, making up 14.9% of the national population, up 0.7 percentage points from 2021 (1). As the population ages and chronic disease and cancer become more common, demand for accessible and competent end-of-life care continues to grow. Personal views of mortality shape emotional and behavioral responses when individuals are confronted with the death of others (2). Nurses often provide direct care to patients near death while also supporting families during the dying process and after bereavement. Fear or avoidance of death may make it difficult for nurses to manage their emotional responses while providing care for dying patients (3). As part of end-of-life care training, death education helps nurses build knowledge about death and improve their ability to respond to death-related care situations (4). Existing evidence indicates that death education may ease death anxiety, improve attitudes toward terminal care, and help nurses feel more prepared to manage death-related situations (5, 6).

In 1986, the American Medical Association (AMA) recommended the inclusion of death education in healthcare professional training. Although death- and palliative-care education is increasingly recognized as important for health-professional training, recent Chinese studies continue to show limited exposure to such training and substantial educational needs among medical and nursing students (7). The End-of-Life Nursing Education Consortium (ELNEC) provides a well-known structured curriculum for improving end-of-life care competence in nursing students and clinical nurses (8). ELNEC-based programs have subsequently been implemented in countries including Germany, Canada, and Singapore (9–11). In Mainland China, traditional reluctance to discuss death has slowed the development of formal death education. However, growing attention to palliative care and care across the life course has encouraged the development of death education programs based on different educational and theoretical models.

In China, Community Health Service Centers (CHSCs) deliver much of the frontline primary care work, including routine care, health education, and preventive services (12). Community nurses are often involved in caring for patients with advanced illness, advising families, and discussing end-of-life issues (13). Their views on death and confidence in handling death-related situations can shape the care they deliver. Yet many community nurses have few opportunities for specialized end-of-life care training, making targeted continuing education necessary. Using the Knowledge-Attitude-Practice framework as its organizing logic, the program focused on three areas: knowledge about death and dying, orientation toward death-related care, and preparation for end-of-life communication and advance care planning (14).

Nurse training may incorporate diverse active-learning strategies, such as flipped classrooms, simulation-based skills demonstrations, case-based learning, group discussions, and problem-based learning (15, 16). However, heavy workloads and scheduling constraints can make prolonged face-to-face training difficult for practicing nurses (17). Mobile health (mHealth) provides a more flexible means of delivering continuing professional education through smartphones and other digital devices, with virtual and augmented reality also being used in some nursing programs (18, 19). In China, standardized death education remains limited in nursing curricula, particularly for professionals working in primary care. WeChat, a widely used mobile communication platform, allows educational materials to be delivered and reviewed without requiring attendance at a fixed time or location. Therefore, we designed a death education program for community nurses, delivered it through WeChat, and assessed changes in death attitudes and coping ability after the training.

## 2 Materials and methods

### 2.1 Study design and ethical considerations

This was a prospective, multicenter, single-group before-and-after study. All participants received the WeChat-based death education training program and completed the same outcome measures before and after the two-month intervention. No parallel control group was included; therefore, the design was used to evaluate within-participant changes rather than to establish definitive causal effects. Ethical approval was obtained from the Ethics Committee of West China Hospital, Sichuan University before the study began (No. 210 of 2023). The trial was registered in the China Clinical Trial Registry (ChiCTR2300073740; 20 July 2023). Reporting followed the Transparent Reporting of Evaluations with Nonrandomized Designs (TREND) statement (20). Before data collection, all participants gave written informed consent. To protect anonymity, questionnaires were labelled with study codes instead of personal identifiers. Although the WeChat backend recorded aggregate reading and engagement metrics, individual questionnaire responses were not linked to identifiable WeChat accounts in the analysis. Participants were told that joining the study was voluntary and that declining would have no effect on their employment, access to training, or professional appraisal.

Potential biases were considered during interpretation. Because the same self-report instruments were administered twice within a two-month period, memory and sensitization bias could not be fully excluded. The trackable nature of the WeChat platform may also have introduced a Hawthorne effect. In addition, maturation and external clinical experiences during the intervention period may have influenced posttest responses. These issues are acknowledged as limitations of the single-group before-and-after design.

### 2.2 Sample and participants

The study took place between October and December 2023 in 10 community health centers of comparable size in Chengdu, Sichuan Province, China. A purposive cluster sampling method was employed. Based on established cooperative relationships, two centers were purposively selected from each of the five major urban districts in Chengdu (Wuhou, Jinjiang, Jinniu, Qingyang, and Chenghua) to ensure geographical representation. Within these selected centers, all eligible nurses were invited to participate.

The required sample size was estimated for a paired pre-post comparison within one group. According to a previous study (21), the “fear of death” dimension of the Death Attitude Profile-Revised (DAP-R) was selected as the primary outcome. The expected mean difference was estimated at 0.405 with a standard deviation of 0.658. With a two-sided *α* of 0.05 and 90% statistical power, the estimated minimum sample size was 28 participants. To accommodate a potential attrition rate of 20%, a minimum of 34 participants was deemed necessary. The final analysis included 129 nurses who completed both the pre- and post-assessments. Based on the observed primary outcome data, the paired mean difference for fear of death was 0.343, the standard deviation of paired differences was 0.401, Cohen’s dz. was 0.856, and the achieved power remained above 99.9% at a two-sided *α* of 0.05.

Eligible participants were registered nurses employed as regular staff in the participating community health centers. They had worked there continuously for at least 6 months, were engaged in routine nursing work during the study period, were able to complete the full training program and both assessments, and provided voluntary consent. Nurses were excluded if they had previously received systematic death education, hospice or palliative care training, or other death-related training before or during the study, or if they were on extended sick or injury leave during the study period.

### 2.3 Data collection tools

#### 2.3.1 Demographic characteristics

Participants completed a demographic questionnaire covering sex, age, education, professional title, nursing experience, religious belief, and prior death-related experiences.

#### 2.3.2 Death attitude profile-revised (DAP-R)

Nurses’ death attitudes were measured with the Death Attitude Profile-Revised (DAP-R), developed by Fry and Wong (22) and later adapted for Chinese use by Tang et al. (23). The 32-item scale includes five subscales: fear of death, death avoidance, natural acceptance, approach acceptance, and escape acceptance. Fear and avoidance reflect negative responses to death, while the three acceptance dimensions capture different ways of understanding death: as a natural event, as entry into an afterlife, or as relief from suffering. Items are rated on a 5-point scale from “strongly disagree” to “strongly agree.”

Because the five DAP-R dimensions measure different aspects of death attitude, no total DAP-R score was used. Dimension scores were expressed as mean item scores, calculated by summing the items within each dimension and dividing by the number of items. A higher score indicates stronger endorsement of that specific attitude, so the dimensions should be interpreted separately. In the Chinese version, Cronbach’s alpha values for the five dimensions ranged from 0.575 to 0.832 (24).

#### 2.3.3 Coping with Death Scale (CDS)

The Coping with Death Scale (CDS) was developed by Bugen (25) in 1980 to assess the effect of death education training. Robbins later reported strong reliability for the scale, with a Cronbach’s alpha of 0.93 (26). The Chinese version was translated by Tseng (27) and has been used in caregiver-related studies. The Chinese CDS includes 30 items across eight death-coping domains, such as self-acceptance, coping confidence, funeral-related tasks, loss coping, life reflection, and discussion of death. Each item is rated from 1 to 7. In this study, CDS results were reported as mean item scores for each dimension and for the overall scale to allow comparison across dimensions. Higher mean item scores represent stronger perceived coping ability with death. The Chinese CDS has shown good reliability and validity among nurses in China.

### 2.4 Interventions

#### 2.4.1 Construction of the content of death education training

In developing the training content, we conducted literature research. We used the basic framework of the Death Education Needs Scale, compiled by Shen (28), and the European and American expert consensus on pre-established medical care programs (29). We combined this with discussions from the research group to form the initial draft of the training content. We then utilized the expert consultation method to refine the initial draft further. The core training content was finalized after two expert consultation rounds and repeated review within the research team.

#### 2.4.2 Delphi expert panel and consultation procedure

Experts were invited if they had worked in a relevant field for at least 10 years, held at least a bachelor’s degree, had an associate-senior or senior professional title, and were familiar with death education. Seven experts participated in the consultation, representing clinical nursing, nursing education, nursing management, palliative care, and clinical medicine.

The consultation questionnaire contained two sections. In the first section, experts scored the importance of each proposed training item from 1 (“very unimportant”) to 5 (“very important”) and provided suggestions on whether items should be added, removed, or revised.

The second collected expert characteristics, familiarity with the topic, and judgement criteria. Between July and September 2023, two electronic consultation rounds were carried out by e-mail or WeChat. After round one, the research team reviewed the experts’ comments, amended the item pool, and sent out the revised questionnaire. Consultation ended after the second round when the expert opinions had reached acceptable consensus.

#### 2.4.3 Delphi data analysis and quality control

Expert characteristics were summarized using mean and standard deviation or frequency and percentage, as appropriate. Item importance was described by the mean score, standard deviation, coefficient of variation (CV), and full-score rate. Expert participation was described by the questionnaire return rate. Expert authority coefficient (Cr) was calculated as Cr = (Ca + Cs)/2, combining the judgement-basis coefficient (Ca) and familiarity coefficient (Cs). Expert agreement was assessed using Kendall’s W and the chi-square test. Items were retained when the mean importance score was at least 3.5, the standard deviation was no greater than 1.0, and the CV was no greater than 0.25, while qualitative comments were considered by the research team. Two researchers independently entered and checked the consultation data.

#### 2.4.4 Death education training intervention program

“The Note of Life” WeChat Official Account was established as the primary channel for delivery. Experts record lectures, edit them into short videos, and add pictures and text to create related resources. These are uploaded to the WeChat public platform and shared on a regular basis. The backend includes a push function and a view count, allowing each new content release to be automatically pushed to followers. The number of readers is displayed to show whether learners have engaged with the content. Users can request relevant information from the WeChat public platform, enabling learners to revisit specific content whenever needed. A WeChat training group was created, which included community nurses, and was followed by the public account. Training materials were released in scheduled batches two to three times per week throughout the two-month intervention.

### 2.5 Statistical analysis

Data entry and preliminary management were performed using Microsoft Excel 2016, while all statistical analyses and data visualizations were conducted using Python 3.11.7. Categorical data were presented as counts and percentages.

Psychometric outcomes were summarized using both *mean ± standard deviation (SD)* and *median (interquartile range, IQR)*. The *Mean ± SD* was retained to allow comparison with previous studies, whereas *median (IQR)* was added to provide an appropriate descriptive summary for outcomes with non-normal distributions. For paired outcomes, the *median (IQR)* of change scores was also reported. The *Shapiro–Wilk* test and skewness coefficients were explicitly utilized to evaluate the normality of pretest scores, posttest scores, and paired difference scores. Because the distributions of most paired differences were significantly non-normal, the changes from pretest to posttest were evaluated using the non-parametric Wilcoxon signed-rank test. Exact *p* values were provided when obtainable. A two-tailed significance threshold of 0.05 was applied for all statistical tests.

## 3 Results

### 3.1 Delphi expert consultation results

Seven experts participated in both consultation rounds. Each expert held at least an associate-senior professional title. Their mean age was 53.00 ± 5.07 years, and their average professional experience was 33.00 ± 6.22 years. In each round, all seven questionnaires were returned and valid, yielding a 100% response rate.

In round one, Ca, Cs, and Cr were 0.957, 0.886, and 0.922, respectively. In round two, these values were 0.971, 0.886, and 0.929. Kendall’s W was 0.499 for primary indicators and 0.331 for secondary indicators in the first round (*chi-square* = 20.970, *p* = 0.002; *chi-square* = 67.157, *p* < 0.001, respectively). Kendall’s W was 0.519 for primary indicators and 0.429 for secondary indicators (*chi-square* = 14.526, *p* = 0.006; *chi-square* = 90.023, *p* < 0.001, respectively) in the second round, indicating statistically significant coordination of expert opinions.

Following the first-round ratings and qualitative suggestions, items were deleted, merged, added, or reworded. In round two, mean importance scores were between 3.71 and 5.00, while CVs ranged from 0.00 to 0.20, showing that experts rated the items highly and with limited variation. After two rounds of consultation and research-team review, the final death education program comprised five primary indicators and 32 secondary indicators, as presented in Table 1.

**Table 1:** The content for death education training.

Module	Primary indicators	Secondary indicators
Module 1	Death and death-education overview	1–1 Concept of death and dying 1–2 Disease and death 1–3 Death and end-of-life care 1–4 Concept and connotation of dignified death 1–5 Concept of euthanasia and related ethics 1–6 Dignified death vs. euthanasia 1–7 Definition of death education 1–8 Current status and development of death education (domestic & international) 1–9 Importance of death education for the nursing profession 1–10 Meaning of life
Module 2	Philosophical, religious & folk views on death & funeral issues	2–1 Philosophical perspectives on death 2–2 Death perspectives in Buddhism, Christianity, etc. 2–3 Folk customs regarding death 2–4 Funeral rites and services
Module 3	Palliative care	3–1 Origin and development of palliative (hospice) care 3–2 Pain and medicine from the perspective of palliative care 3–3 Concept, origin, and development of palliative care 3–4 Comfort Care in Palliative Care 3–5 Humanistic Care in Symptom Management 3–6 Differentiation of Concepts: Palliative Care, Hospice Care, and End-of-Life Care
Module 4	Advance care planning	4–1 Concept and Significance of Advance Care Planning 4–2 Components and Implementation of Advanced Care Planning 4–3 Advance Directives (ADs): Concepts, Types, and Content 4–4 Living Will: Concept and Content 4–5 Relationship among Living Will, Advance Directives, and Advance Care Planning 4–6 ‘Do Not Resuscitate’ (DNR) Orders in the ICU 4–7 Communication Role of Nursing Staff in Advanced Care Planning
Module 5	Care for dying and near-death patients	5–1 Psychological Characteristics and Needs of Near-Death Patients 5–2 Psychological Characteristics and Needs of Families of Near-Death Patients 5–3 Symptom Management and Nursing Care for Near-Death Patients 5–4 Communication Strategies with Near-Death Patients and Their Families 5–5 Personal Psychological Adjustment for Nurses Caring for Near-Death or Dying Patients

ADs, advance directives; DNR, do-not-resuscitate; ICU, intensive care unit.

### 3.2 General conditions of community nurses

The analysis included 129 community nurses who completed both assessments. Among them, four were male (3.1%) and 125 were female (96.9%). The mean age was 30.57 ± 8.32 years. Participant characteristics are shown in Table 2.

**Table 2:** Participants’ demographics and clinical-related experience (*N* = 129).

Variant	Categorization	*n* (%) or mean ± SD
Age, years		30.57 ± 8.32
Sex	Male	4 (3.1)
	Female	125 (96.9)
Marital status	Unmarried	62 (48.1)
	Married	65 (50.4)
	Divorce	2 (1.6)
Educational background	Master’s degree and above	2 (1.6)
	University undergraduate course	62 (48)
	College and below	65 (50.4)
Professional title	Associate senior nurse or senior nurse	5 (3.9)
	Supervisor nurse	34 (26.4)
	Nurse practitioner	45 (34.9)
	Assistant nurse	45 (34.9)
Years of experience	<5 years	55 (42.6)
	5–10 years	29 (22.5)
	>10 years	45 (34.9)
Religious belief, yes		9 (7.0)
Hospitalization experience, yes		71 (55.0)
Bereavement experience, yes		79 (61.2)
Attendance at funerals, yes		120 (93.0)
Exposure to dying patients, yes		108 (83.7)
Family discussion of topics related to death	No discussion	28 (21.7)
	Occasional discussion	75 (58.1)
	Reactive/Forced discussion	14 (10.9)
	Regular public discussions	12 (9.3)

Data are presented as *n* (%) unless otherwise indicated. Age is presented as mean ± standard deviation (SD).

### 3.3 Comparison of DAP-R scores before and after training

**Figure 1:**
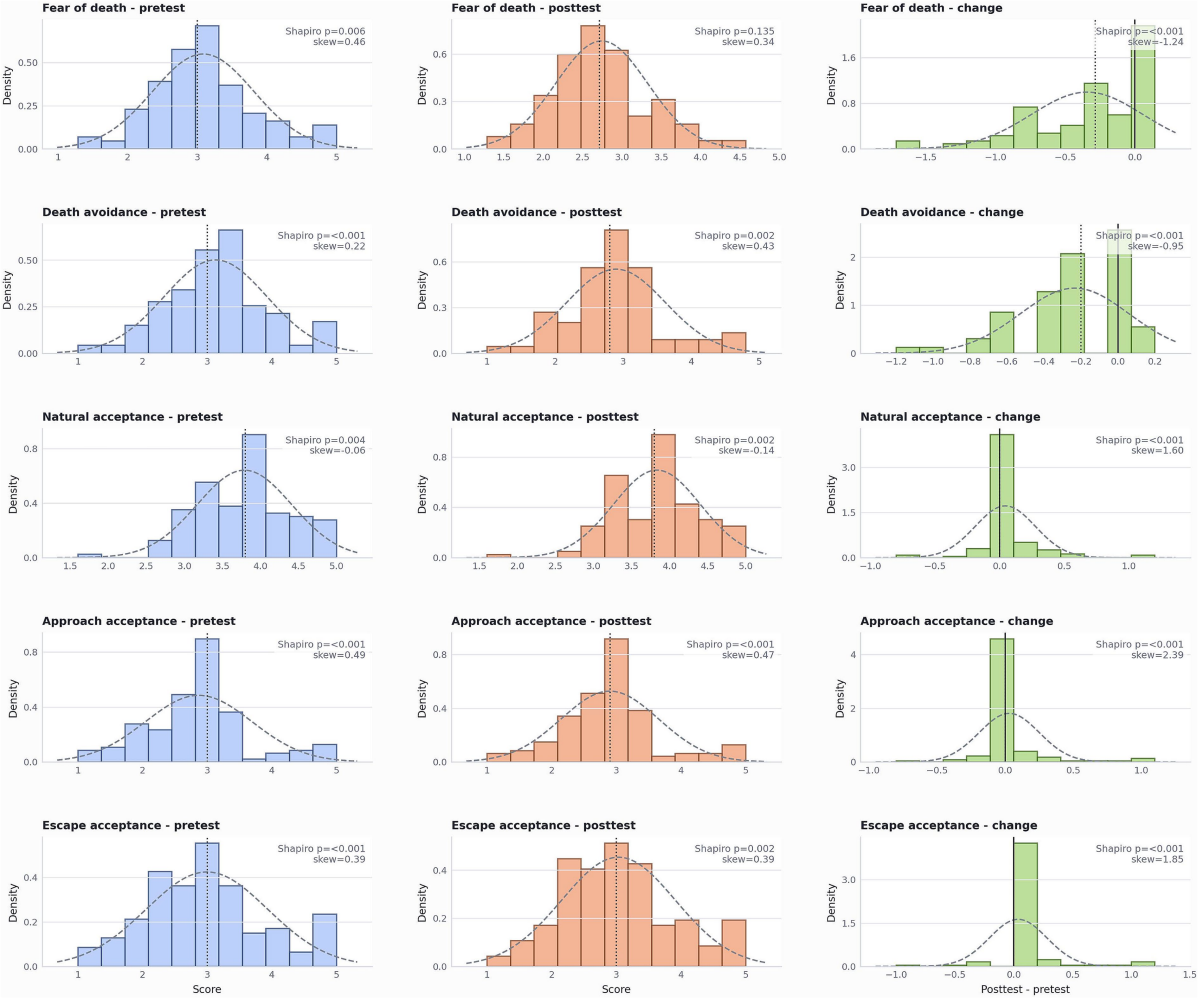
Distribution of DAP-R pretest, posttest, and paired change scores. Shapiro–Wilk *p* values and skewness are shown for each distribution. DAP-R, Death Attitude Profile-Revised; p, Shapiro–Wilk normality test probability value; skew, skewness coefficient. Change scores were calculated as posttest minus pretest. Blue, orange, and green represent pretest, posttest, and change-score distributions, respectively. The dashed curve represents the reference normal distribution.

The Shapiro–Wilk tests and skewness coefficients indicated that most pretest, posttest, and paired difference distributions deviated from normality, especially the paired change scores. Therefore, Wilcoxon signed-rank tests were used for the pre-post comparisons. After the intervention, scores for fear of death and death avoidance on the DAP-R decreased significantly (both *p* < 0.001), suggesting less negative orientation toward death. No significant changes were found in natural acceptance (*p* = 0.054), approach acceptance (*p* = 0.339), or escape acceptance (*p* = 0.082). The detailed data are presented in Table 3, and the distribution patterns are shown in Figure 1.

**Table 3:** Comparison of DAP-R dimension scores before and after training (N = 129).

DAP-R dimension	Pretest mean ± SD	Pretest median (IQR)	Posttest mean ± SD	Posttest median (IQR)	Change median (IQR)	*Wilcoxon Z*	*p*	*Effect size r*
Fear of death	3.09 ± 0.73	3.00 (2.71–3.43)†	2.74 ± 0.58	2.71 (2.43–3.00)	−0.29 (−0.57–0.00)†	−7.922	<0.001	0.70
Death avoidance	3.13 ± 0.80	3.00 (2.80–3.40)†	2.89 ± 0.72	2.80 (2.40–3.20)†	−0.20 (−0.40–0.00)†	−7.408	<0.001	0.65
Natural acceptance	3.79 ± 0.62	3.80 (3.40–4.20)†	3.83 ± 0.57	3.80 (3.40–4.20)†	0.00 (0.00–0.00)†	−1.931	0.054	0.17
Approach acceptance	2.87 ± 0.82	3.00 (2.40–3.10)†	2.90 ± 0.76	2.90 (2.50–3.10)†	0.00 (0.00–0.00)†	−0.956	0.339	0.08
Escape acceptance	3.00 ± 0.94	3.00 (2.40–3.40)†	3.03 ± 0.88	3.00 (2.40–3.40)†	0.00 (0.00–0.00)†	−1.739	0.082	0.15

Data are presented as mean ± standard deviation (SD). DAP-R, Death Attitude Profile-Revised; Z, standardized Wilcoxon signed-rank test statistic. Negative *Z* values indicate lower posttest ranks relative to pretest ranks according to the specified comparison direction; IQR, interquartile range; †, non-normal distribution by the Shapiro–Wilk test (*p* < 0.05). Change scores were calculated as posttest minus pretest.

### 3.4 Comparison of CDS scores before and after training

**Figure 2:**
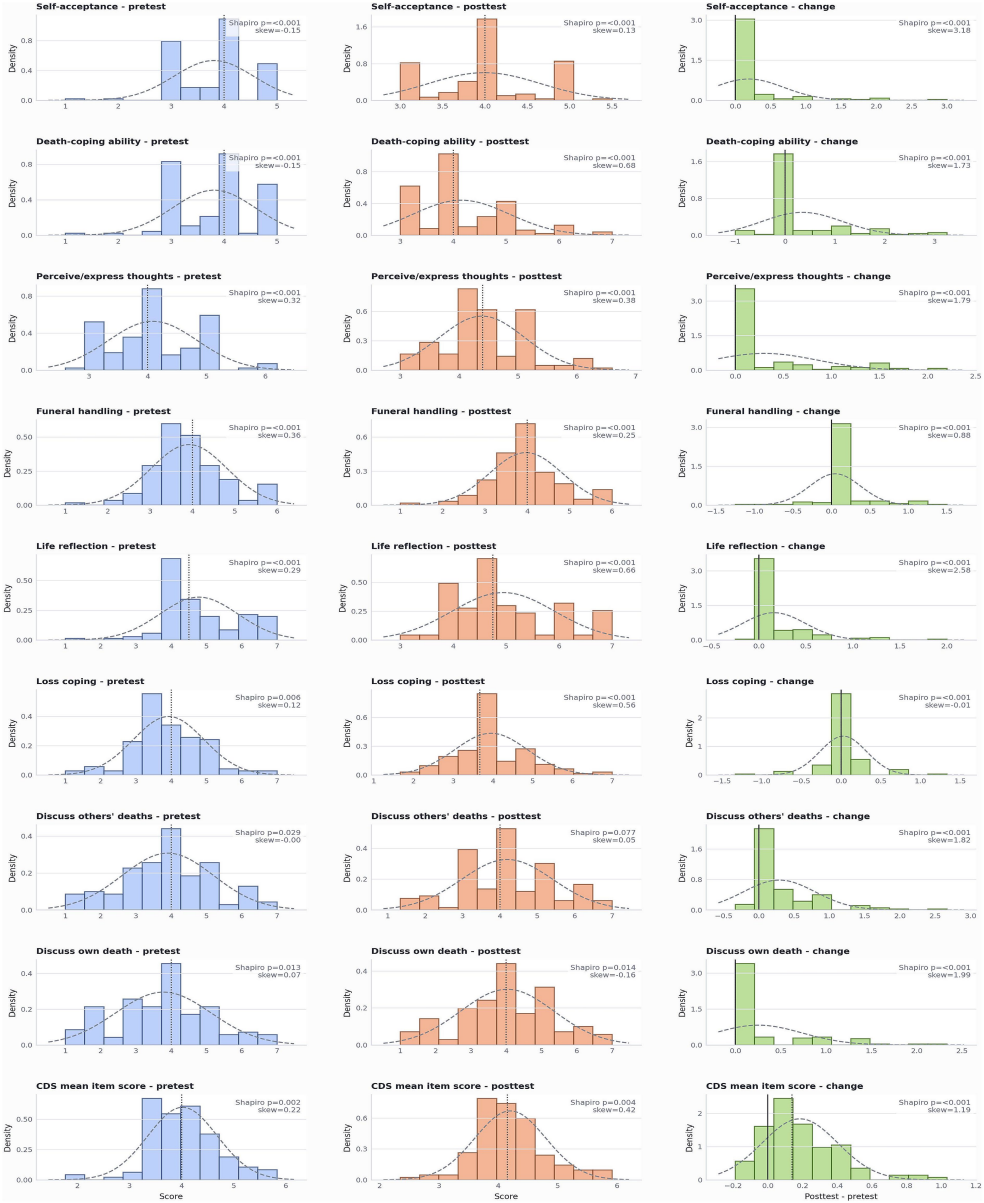
Distribution of CDS pretest, posttest, and paired change scores. Shapiro–Wilk p values and skewness are shown for each distribution. CDS, Coping with Death Scale; p, Shapiro–Wilk normality test probability value; skew, skewness coefficient. Change scores were calculated as posttest minus pretest. Blue, orange, and green represent pretest, posttest, and change-score distributions, respectively. The dashed curve represents the reference normal distribution.

The CDS mean item score and most CDS dimension scores improved after the WeChat-based death education training. Wilcoxon signed-rank tests indicated significant post-training increases in self-acceptance of death, death-coping ability, perception and expression of death-related thoughts, life reflection ability, discussion of others’ deaths, discussion of one’s own death, and the overall CDS mean item score (all *p* < 0.001). No significant differences were observed for funeral handling ability (*p* = 0.119) or coping with loss (*p* = 0.141). Detailed results are provided in Table 4, and the score distributions are shown in Figure 2.

**Table 4:** Comparison of CDS mean item scores before and after training (*N* = 129).

CDS dimension	Pretest (mean ± SD)	Pretest median (IQR)	Posttest (mean ± SD)	Posttest median (IQR)	Change median (IQR)	*Wilcoxon Z*	*p*	*Effect size r*
Self-acceptance of death	3.80 ± 0.75	4.00 (3.00–4.00)†	3.99 ± 0.66	4.00 (3.75–4.25)†	0.00 (0.00–0.00)†	−4.137	<0.001	0.36
Death-coping ability	3.81 ± 0.79	4.00 (3.00–4.00)†	4.15 ± 0.90	4.00 (3.50–4.75)†	0.00 (0.00–0.50)†	−4.418	<0.001	0.39
Perceive/express thoughts about death	4.09 ± 0.76	4.00 (3.60–4.80)†	4.39 ± 0.72	4.40 (4.00–5.00)†	0.00 (0.00–0.40)†	−5.447	<0.001	0.48
Funeral handling ability	3.92 ± 0.90	4.00 (3.50–4.50)†	3.97 ± 0.86	4.00 (3.50–4.50)†	0.00 (0.00–0.00)†	−1.561	0.119	0.14
Life reflection ability	4.78 ± 1.11	4.50 (4.00–5.50)†	4.95 ± 0.97	4.75 (4.25–5.50)†	0.00 (0.00–0.25)†	−5.179	<0.001	0.46
Loss coping ability	3.93 ± 1.00	4.00 (3.33–4.33)†	3.95 ± 0.92	3.67 (3.33–4.33)†	0.00 (0.00–0.00)†	−1.473	0.141	0.13
Discuss others’ deaths	3.90 ± 1.30	4.00 (3.00–4.67)†	4.19 ± 1.22	4.00 (3.33–5.00)	0.00 (0.00–0.33)†	−5.717	<0.001	0.50
Discuss one’s own death	3.78 ± 1.35	4.00 (3.00–4.67)†	4.03 ± 1.32	4.00 (3.33–5.00)†	0.00 (0.00–0.33)†	−5.247	<0.001	0.46
CDS mean item score	4.02 ± 0.67	4.00 (3.53–4.40)†	4.20 ± 0.60	4.15 (3.80–4.48)†	0.14 (0.04–0.30)†	−8.211	<0.001	0.72

Data are presented as mean ± standard deviation (SD). CDS, Coping with Death Scale; *Z*, standardized Wilcoxon signed-rank test statistic; IQR, interquartile range; †, non-normal distribution by the Shapiro–Wilk test (*p* < 0.05). Change scores were calculated as posttest minus pretest. Negative *Z* values indicate lower posttest ranks relative to pretest ranks according to the specified comparison direction.

## 4 Discussion

This study examined changes in community nurses’ attitudes toward death and death-coping ability after a two-month WeChat-based death education program. Participants reported lower fear and avoidance of death after training, along with improvements in several perceived death-coping domains. In contrast, the three acceptance-related dimensions of the DAP-R and the CDS dimensions concerning funeral handling and coping with loss did not change significantly. Taken together, the findings suggest that the program was more effective in addressing fear, avoidance, and perceived readiness than in changing established beliefs about death or skills that depend heavily on clinical experience.

Natural acceptance was the highest DAP-R dimension both before and after training. Similar findings have been reported among nurses in emergency, hospital, and primary-care settings (30, 31). Recognizing death as a natural event does not necessarily prevent fear or avoidance during encounters with dying patients. Nurses’ responses to death are influenced by personal beliefs, previous exposure, workplace culture, and the emotional demands of care (32). The decrease in fear and avoidance in this study is consistent with earlier nurse-education studies that reported improvements in death-related attitudes after structured training (3, 11). However, Hao et al. (33) found no significant change in DAP-R scores after a shorter mobile learning intervention. Program duration and content may have contributed to the different findings, although this cannot be established without a control group. The absence of change in natural, approach, and escape acceptance may also reflect the relative stability of culturally and spiritually grounded beliefs. Future training should combine factual content with guided reflection and discussion of clinical cases, as knowledge alone may be insufficient to change these dimensions (34).

Most CDS dimensions improved after training, suggesting greater perceived ability to think and talk about death and to manage death-related situations. Death competence has been associated with professional experience, self-efficacy, and opportunities to examine personal responses to death (26, 32). Improvements in coping after structured death education have also been reported among oncology nurses (35). Nevertheless, the practical importance of the present change should not be overstated. The CDS mean item score increased by 0.18 points on a seven-point scale. Although the rank-based effect size was large, no minimally important difference has been established for the CDS mean item score; statistical significance alone therefore does not demonstrate a meaningful change in clinical practice (36). Funeral handling and coping with loss did not improve significantly. These abilities involve local procedures, grief support, emotional regulation, and experience with bereaved families (37, 38). They are unlikely to develop through online theoretical instruction alone. Scenario-based exercises, supervised communication, and grief-counselling practice should be considered in future programs.

WeChat provided flexible access and allowed nurses to review the materials around their work schedules. Smartphone and website-based learning have been used successfully in nurses’ continuing education, particularly for knowledge and specific clinical tasks (17, 32). WeChat has also been used in nurse in-service training in China. These advantages make it a practical channel for introductory death education in community settings. However, reading or viewing a resource does not show that the learner understood it or can apply it in practice. A blended program would be more appropriate for complex end-of-life competencies: WeChat could deliver core knowledge, while workshops, role-play, and supervised practice could develop communication and care skills. Communication-focused curricula and active learning approaches offer useful models for this component (9, 39). Future studies should assess engagement, knowledge retention, observed performance, and longer-term outcomes rather than relying only on self-reported attitudes and coping.

### 4.1 Limitations

Several limitations should be considered. First, the study used a single-group pretest-posttest design without a parallel control group. Changes observed during the two-month study period therefore cannot be attributed exclusively to the WeChat-based program. History effects, natural changes in participants’ coping responses, and changes resulting from routine clinical experience may also have contributed to the posttest scores. Because information on intervening clinical events was not systematically collected, these influences could not be adjusted for in the analysis. Second, the same self-reported DAP-R and CDS instruments were administered at both assessments. Previous exposure to the questionnaire items may have affected participants’ posttest responses through recall, sensitization, or greater awareness of the study objectives. Because the outcomes were self-reported, responses may have been influenced by social desirability and may not correspond to actual clinical behavior. Accordingly, the findings should be interpreted as within-participant changes associated with the intervention period rather than definitive evidence of an intervention effect. Future studies should include a concurrent control group, record relevant clinical exposures during follow-up, use additional measurement points, and incorporate objective or performance-based outcomes.

## 5 Conclusion

This study developed a WeChat public platform-based death education program for community nurses through expert consultation and evaluated changes before and after training. The program was followed by lower fear and avoidance of death and by gains in several death-coping domains. Given the single-group before-and-after design, these findings should be interpreted cautiously. The program may serve as a feasible model for community nurse continuing education, but controlled studies with longer follow-up are needed before broad causal conclusions can be drawn.

## Data Availability

The original contributions presented in the study are included in the article/supplementary material, further inquiries can be directed to the corresponding author.
